# What Could Be Future Scenarios?—Lessons from the History of Public Health Surveillance for the Future ––A keynote address presented at the 8th World Alliance for Risk Factor Surveillance (WARFS) Global Conference on October 30, 2013, Beijing, China

**DOI:** 10.3934/publichealth.2015.1.27

**Published:** 2015-03-09

**Authors:** Bernard C.K. Choi

**Affiliations:** 1 Health Promotion and Chronic Disease Prevention Branch, Public Health Agency of Canada, Government of Canada, Ottawa, Ontario, Canada; 2 Dalla Lana School of Public Health, University of Toronto, Toronto, Ontario, Canada; 3 Injury Prevention Research Center, Shantou University Medical College, Shantou, China

**Keywords:** public health surveillance, history, lessons learned, future scenarios, public health, health promotion, disease prevention

## Abstract

This article provides insights into the future based on a review of the past and present of public health surveillance—the ongoing systematic collection, analysis, interpretation, and dissemination of health data for the planning, implementation, and evaluation of public health action. Public health surveillance dates back to the first recorded epidemic in 3180 BC in Egypt. A number of lessons and items of interest are summarised from a review of historical perspectives in the past 5,000 years and the current practice of surveillance. Some future scenarios are presented: exploring new frontiers; enhancing computer technology; improving epidemic investigations; improving data collection, analysis, dissemination and use; building on lessons from the past; building capacity; and enhancing global surveillance. It is concluded that learning from the past, reflecting on the present, and planning for the future can further enhance public health surveillance.

## 1. Introduction

The term “surveillance”, derived from the French roots, *sur* (over) and *veiller* (to watch) [1], is defined in the dictionary as the “close and continuous observation of one or more persons for the purpose of direction, supervision, or control” [2]. For the purpose of this article, public health surveillance is defined as “the ongoing systematic collection, analysis, interpretation and dissemination of health data for the planning, implementation and evaluation of public health action” [3]. The objective of this article is to provide insights into the future, based on a review of the past and present of public health surveillance.

## 2. Materials and method

The review on the past, present and future of surveillance was based on an extensive consultation of relevant documents including manuscripts, books, and reports, both in print form and on the internet. The brief section on the past reviewed major epidemics in human history, historical milestones in the development of public health surveillance, and lessons learned. As much as possible, original historical documents have been reviewed and directly quoted. The brief section on the present reviewed some definitions and components of public health surveillance. The emphasis of this article is on the section on the future which reviewed the literature concerning possible scenarios and directions proposed by various authors for the future development of public health surveillance.

## 3. Results

### 3.1 The past

The first recorded epidemic in human history was “a great pestilence” that occurred in Egypt during the reign of Pharaoh Mempses in the First Dynasty, 3180 BC [4]. Manetho, the Egyptian priest and historian, stated in his list of pharaohs, “*Mempses, for eighteen years. In his reign many portents and a great pestilence occurred*” [5]. The three most devastating epidemics to hit the human race were: "The Plague of Justinian" (AD 541–591) which lasted 50 years, "The Black Death" (1348–1351) which lasted 4 years, and "Spanish Influenza" (1918) which lasted five months [6]. The history of public health surveillance is full of blood, sweat and tears – blood of injury and sacrifice, sweat of hard labour, and tears of setback and failure. Detailed accounts of the historical milestones and lessons learned from the past 5,000 years of human history in surveillance are not described here as they are available elsewhere [3],[4].

It is important to learn lessons from the past, so we can do things better in the present and in the future. For example, one lesson is to continue to systematically collect the three types of information that were included in the historical records of epidemics: health outcomes (e.g. plague, dancing mania, etc.), risk factors (drought, poor sanitation, etc.) and interventions (quarantine of ships, hand washing, etc.) [3]. Another lesson is to enhance the early warning function of surveillance [4]. By the time of the 3^rd^ cholera pandemic which started in India (1846) and ended in North and Central America (1863), the common origin (India) of the pandemic was well recognized (Table 1). A warning system was in place which used the customs service and diplomatic corps to get reports of where infected ships might be coming in order to implement quarantine. This helped alleviate the 4^th^ cholera pandemic which started again in India (1863) [4]. A further lesson is to link surveillance to intervention. This is intuitively the right thing to do but is not always possible. The “Spanish Influenza” (1918) killed 22 million people, more than twice the 10 million deaths caused by World War I (1941–1918). No intervention was available at the time. It was only until 1933, 14 years afterwards, that the virus was isolated, and 1972, 54 years after the epidemic had hit, that the vaccine for influenza was developed [4].

**Table 1.** Four pandemics of cholera (1817–1823, 1826–1837, 1846–1863, 1863–1875) [4].

Duration	Route of cholera pandemic
**6 years**	Calcutta (1817) … Russia (1823)
**11 years**	North East India (1826) … Africa (1837)
**17 years**	India (1846) … North and Central America (1863)
**12 years**	Ganges (1863) … South America (1875)

### 3.2 The present

There are many articles and books on the current principles and practice of public health surveillance so this is not repeated in detail here. Interested readers are referred to consult the reference lists of several review papers on public health surveillance [3],[4].

It is of interest to compare the description of early surveillance activities written by John Graunt in 1662 [7] and the current definition of public health surveillance provided by the World Health Organization (WHO) [8]. In his book “*Natural and political observations made upon the bills of mortality*” published in 1662, Graunt wrote, “*Now having engaged my thoughts upon the Bills of Mortality, and so far succeeded therein, as to have reduced several great confused Volumes into a few perspicuous Tables, and abridged such Observations as naturally flowed from them, into a few succinct Paragraphs … I hoped … to see unto how much profit that one Talent might be improved, beside the many curiosities concerning the waxing and waning of Diseases*” [7]. When broken down into various components, it can be seen that the description of the work of John Graunt corresponds almost exactly to the current WHO definition of public health surveillance (Table 2). Over 350 years, nevertheless, the purpose of public health surveillance has shifted from “curiosities” to “public health practice”.

**Table 2.** Comparison of two public health surveillance definitions in 1600s and 2000s.

Component	John Graunt (1662) [7]	World Health Organization (2015) [8]
Process	Reduced several great confused Volumes (of the Bills of Mortality) into a few perspicuous Tables	Continuous, systematic collection, analysis
Output	Abridged such Observations … into a few succinct Paragraphs	And interpretation of health-related data needed for the …
Purpose	See unto how much profit that one Talent might be improved, beside the many curiosities concerning the waxing and waning of Diseases	Planning, implementation, and evaluation of public health practice

Another item of interest is to compare the similarities of public health surveillance and modern video camera surveillance. In essence, public health surveillance is the ongoing collection, analysis, and interpretation of health data essential to public health practice, closely integrated with timely dissemination of information for intervention. This is analogous to a 24 hour surveillance camera (data collection) under the watchful eyes of guards (data analysis and interpretation) who have telephone access (information dissemination) to the police (intervention) [9].

### 3.3 The future

The future cannot be predicted with certainty but this is the main focus of this article. There are already published articles that present views on public heath surveillance in the future. Some current efforts and activities that can affect future directions are summarized below.

#### 3.3.1 Exploring new frontiers for public health surveillance

Historically, surveillance focused on infectious disease, then broadened to other topics, such as chronic diseases [10], occupational health [11], environmental health [12], hazard surveillance [13], emerging infectious diseases [14], injury control [15], behavioural risk factors [16], events following disasters [17], pharmacosurveillance [18], and firearm-related injury [19]. At this time, mental health and mental illness [20], and obesity [21], are also recognized as domains in public health surveillance. It is expected that further new frontiers will be explored in the future for surveillance [3].

New frontiers mean new challenges and solutions. Take, for example, surveillance activities on obesity. The prevalence of obesity has increased substantially over the past 30 years. Although the spread of obesity in large social networks over extended periods of time is difficult to track, this has been done and reported in a paper “The spread of obesity in a large social network over 32 years” [22]. Moreover, a 3D animation video summarizing the results of tracking a large social network over 3 decades, locating clusters of obese persons and similar clusters of nonobese persons, has been uploaded on YouTube and is interesting to watch (Box 1) [23].

**Box 1. A video showing a 3D animation of the spread of obesity in a large social network over 32 years (1:48) [23].**

Available from: http://www.youtube.com/watch?v=pJfq-o5nZQ4

Uploaded on Feb 4, 2010 by Nicholas Christakis

Source: Christakis NA, Fowler JH. (2007) The spread of obesity in a large social network over 32 years. N Engl J Med357:370-379 [22].

Available from: http://media.timesfreepress.com/docs/2010/01/Obesity_studies_0104.pdf

**Description:** This animation is a dynamic graphic representation of a portion of the Framingham Heart Study social network. Each circle or node represents one person in the dataset. Nodes with red borders are women, and nodes with blue borders are men. The size of each node is proportional to each person's body mass index or BMI which is the weight in kilograms divided by the square of the height in meters. A yellow node represents an obese person with a BMI greater than 30, and a green node represents a nonobese person. A line between 2 nodes represents the social connection between 2 people. Purple lines connecting nodes denote close genetic ties for example parents, children and siblings. Grey lines denote nongenetic relations such as friends and spouses.

Obesity is a multicentric epidemic with many people influencing one another, forming ties in complex ways. The network gets more compact and dense as more ties appear. And the entire network gets heavier over time. Quantitative analyses demonstrate tendencies for obese people and nonobese people to form clusters within the network, so that by the end of the study this clustering can be discerned as in the densely interconnected clusters of obese persons here and here, and the nonobese persons here. The prevalence of obesity appears lower at the periphery of the network, as compared with the center.

#### 3.3.2 Enhancing the use of computer technology in public health surveillance

Computer technology can improve public health information systems which vary from a simple system collecting data from a single source, to electronic systems that receive data from many sources in multiple formats, to complex surveys [24]. New terms like “infodemiology”, “infoveillance”, “datafication”, “dataism”, and “dataveillance” have been coined for the use of informatics methods to analyze queries from the Internet search engines to predict disease outbreaks [25],[26] (Table 3).

**Table 3.** Definitions of new terms in public health informatics.

Term	Definition
**Public health informatics**	The systematic application of information and computer science and technology to public health practice, research and learning [27].
**Infodemiology**	The science of distribution and determinants of information in an electronic medium, specifically the Internet, or in a population, with the ultimate aim to inform public health and public policy [28].
**Infoveillance**	Using infodemiology data for surveillance purposes [28].
**Datafication**	The transformation of social action into online quantified data, thus allowing for real-time tracking and predictive analysis [26].
**Dataism**	An ideology that shows characteristics of a widespread belief in the objective quantification and potential tracking of all kinds of human behavior and sociality through online media technologies, as well as trust in the (institutional) agents that collect, interpret, and share (meta) data culled from social media, internet platforms, and other communication technologies [26].
**Dataveillance**	A form of continuous surveillance through the use of (meta) data [26].

One example showing potential that computer technology will improve the quality, capacity, and effectiveness of public health surveillance is the use of a promising interactive health information technology called “eHealth” [29]. Other technologies include a novel approach for detecting influenza outbreaks using search engine query data. Historical logs of more than 50 million of the most common online Web search queries were analyzed to track influenza-like illness in different areas and regions. There was a high correlation of Google queries (influenza-like illness-related search queries) with the percentage of physician visits in patients with influenza-like symptoms [30].

The power of the Internet cannot be underestimated. For example, The Opte Project (pronounced op-tee), originated from the Latin word opti, meaning “optical”, seeks to make an accurate visual graphical representation of the extent of the Internet [31]. The Opte Project provides links to a 2D visualization of routing paths through a portion of the Internet (Box 2a) [32] and to a video (Movie 4) that shows a 3D animation of the routing paths and IP addresses (Internet Protocol addresses) inside the Internet (Box 2b) [33]. The 2D visual graphical presentation and the 3D video show the tremendous size and power of the Internet.

The power of the Internet goes beyond visualization. Crowdsourcing (the practice of obtaining needed services, ideas, or content by soliciting contributions from a large group of people) is facilitated by the Internet. Crowdsourcing is used by ProMEDmail [34] to maintain a global electronic reporting system for outbreaks of emerging infectious diseases and toxins. It is also used by the World Health Organization to implement the International Health Regulations (IHR) [35]. Use of computer technology and the Internet will continue to contribute to the evolution of public health surveillance.

**Box 2a. A 2D visual graphical presentation from the Opte Project of the various routes through a portion of the Internet [32]**

Available from: http://en.wikipedia.org/wiki/Opte_Project#mediaviewer/File:Internet_map_1024.jpg

**Description:** Partial map of the Internet based on the January 15, 2005 data found on opte.org. Each line is drawn between two nodes, representing two IP addresses (Internet Protocol addresses). The length of the lines is indicative of the delay between those two nodes. This graph represents less than 30% of the Class C networks reachable by the data collection program in early 2005. Lines are color-coded according to their corresponding RFC 1918 allocation as follows: Dark blue: net, ca, us Green: com, org Red: mil, gov, edu Yellow: jp, cn, tw, au, de Magenta: uk, it, pl, fr Gold: br, kr, nl White: unknown

**Box 2b. A videoshowing a 3D animation of the routing paths and IP addresses (Internet Protocol addresses) inside the Internet [33]**

Available from: http://www.opte.org/maps/

[click on movie4.mpeg Dec 8 2003 (879k)]

**Description:** Movie 4 is our first hand made animation. The quality is poor and the runtime is too fast, but it gives someone an idea of what we really can do with this stuff once we have some computing power.

#### 3.3.3 Improving methods of epidemic investigations

New science and technology will continue to improve the approach to epidemiologic outbreak investigations. Rapid technology development in the laboratory has improved diagnostic precision and reduced the time necessary to make a diagnosis. These improvements should continue, for example, to identify pathogens in imported foods at the place of importation and among persons who now travel more extensively and more rapidly around the globe [36]. Statisticians continue to develop new statistical methods that will provide insights through refined data analysis. Improved techniques for training also need to be developed so that the technology of epidemic investigations can be used effectively by public health practitioners [36].

**Figure 1.**
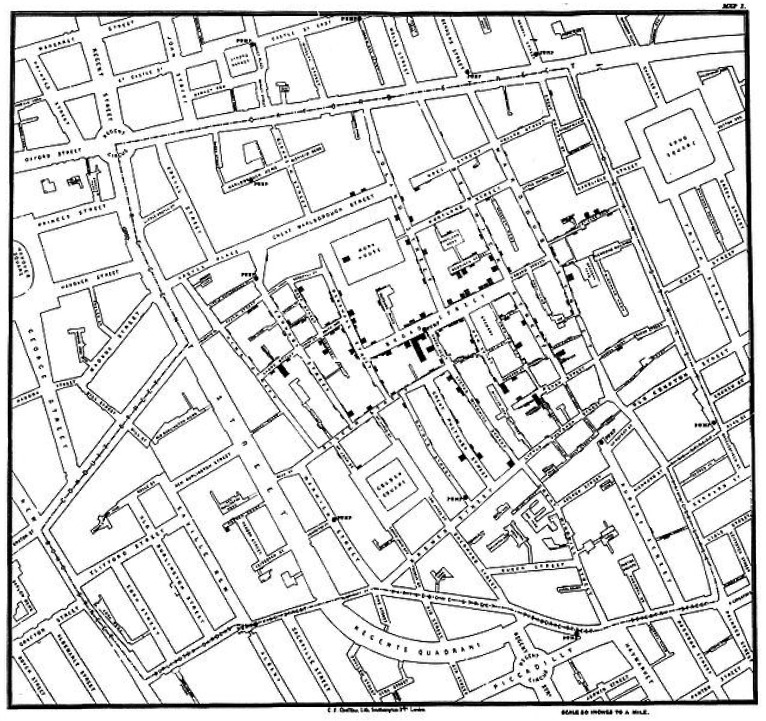
Original map made by John Snow in 1854* [37]. Cholera cases are highlighted in black. (Available from: http://commons.wikimedia.org/wiki/File:Snow-cholera-map-1.jpg) *This image is in the public domain due to its age. Author died in 1858. Material is public domain.

It is of interest to look at advances in graphical presentation methods in the last 160 years, by comparing the original map made in 1854 by John Snow (1813–1858) (Figure 1, Box 3a) [37] on his cholera investigation in London, with the modern 3D version made in 2011 (Box 3b) [38]. The modern 3D map, besides providing a 3-dimensional representation of the number of deaths, looks very similar to the original 2D map made in 1854. It seems there is still room for future improvement in graphical methods.

**Box 3a. Original 2D map made by John Snow in 1854 [37]**

Available from: http://commons.wikimedia.org/wiki/File:Snow-cholera-map-1.jpg

**Description:** Original map made by John Snow in 1854. Cholera cases are highlighted in black.

**Author:** John Snow

**Permission (Reusing this file):** This image is in the public domain due to its age. Author died in 1858, material is public domain. This image (or other media file) is in the public domain because its copyright has expired. This applies to Australia, the European Union and those countries with a copyright term of life of the author plus 70 years.

**Box 3b. A 3D visualization made in 2011of analysis of the original map made by John Snow [38]**

Available from: http://geolutions.wordpress.com/

**Description:** 3D visualization of analysis. Blue flags represent pumps for drinking water, red bars represent deaths at that address.

#### 3.3.4 Improving methods of data collection

Telephone surveys have been a powerful tool for data collection. However, the use of telephone-based random-digit-dialling methods in public health surveys and surveillance is now at a crossroads [36]. Rapid changes in telecommunication, declines in participation rates, increases in the required level of effort and associated costs are becoming key challenges for telephone surveys. Mixed-mode surveys, that utilize more than one mode of data collection, are being developed to tackle some of these problems.

Other challenges in data collection include population mobility. mHealth (also written as m-health), or mobile health, is the practice of medicine and public health supported by mobile devices [39]. Fibit Tracker (manufactured by Fibit Inc.), a wireless-enabled wearable device, can be used to measure data such as the number of steps walked, quality of sleep, and other personal metrics. These methods can help resolve some of the problems in population mobility.

It will be important to continue to improve existing methodology and develop new cost-effective and more accurate data collection methodologies for the future. Future surveys should collect physical measurements as part of its ongoing operation, as several studies have examined the limitations of self-reported survey data and their impact on estimates such as obesity and blood pressure using national surveys [40],[41].

#### 3.3.5 Improving methods of epidemiologic and statistical analysis

A renewed activity associated with public health surveillance is that of the methods of epidemiologic and statistical analysis [42]. Since the 1980s, applications and methods of time series methods have enabled more meaningful analysis and interpretation of data collected from surveillance [43]. More sophisticated techniques such as geographical and spatial methods and space-time monitoring will continue to be applied to public health surveillance as they are developed [44]. Postsurvey adjustments are becoming an increasingly important means of maintaining the representativeness of survey data. New weighting methods have been developed for adjusting the data for sex, age, race, education, marital status and telephone coverage [45], and for non-telephone coverage [46].

Time series methods produce excellent line charts, and are particularly good for prediction and forecasting (Box 4) [47]. The line charts from time series provide the best-fit line and other lines by applying different filters to achieve different purposes. They will have a wider application in future as a useful tool for public health surveillance.

**Box 4. A 2D graphical representation of results from a time series [47]**

Available from: http://en.wikipedia.org/wiki/Time_series#mediaviewer/File:Random-data-plus-trend-r2.png

**Description:** Time series: random data plus trend, with best-fit line and different applied filters.

#### 3.3.6 Improving methods of information dissemination

Since late 19th century, the dissemination of surveillance information generally has been done by “weekly reports” of diseases of critical health or strategic importance [48]. Until recently, surveillance information was disseminated as written documents published periodically by government agencies [42].

Although printed paper reports will continue to be produced, there is a need to explore new methods of information dissemination, such as paperless or electronic media [49]. Associated with ready electronic access to detailed personal information from surveillance are ethical and legal concerns that might constrain access to data of potential public health importance [50].

#### 3.3.7 Improving use of surveillance information by decision makers

Perhaps most importantly, surveillance information should be used more by decision makers [51]. It must, however, be recognized that while public health surveillance is the cornerstone of public health practice [52], it is not the only source of information for evidence-based public health [53], as surveillance is only one element in the package of evidence to influence healthy public policies [51]. There are at least 5 tools/processes for decision makers on public health actions: meta-analysis, risk assessment, economic evaluation, public health surveillance, and expert panels/consensus conferences [54]. In addition to scientific evidence, policy making is also based on values, emotions, and the wishes of interest groups [55].

Numerous methods have been proposed to bridge the gap between knowledge producers (researchers) and knowledge users (decision makers). These include incentives for researchers and decision makers to work more closely together, and introduction of “knowledge brokers” [55],[56]. In a way, the working together of researchers and decision makers is analogous to a gearbox in a machine. Several online animations of gears and gearboxes show how gears (researchers and decision makers) can engage each other in a large number of different ways (Box 5a) [57], how a third gear (a knowledge broker) can make two engaging gears (a researcher and a policy maker) turn in the same direction (Box 5b) [58], and why the linear speed at the outer parts of two gears (a fast rotating small gear and a slow rotating large gear) must be the same – researchers and policy makers can work separately on their spheres of activities but when working together they must be synchronized (Box 5c) [59].

There are emerging practices to improve the use of surveillance information by decision makers. One example is health impact assessment (HIA). HIA is a practical approach used to judge the potential health effects of a proposed policy or program on a population [60]. Recommendations are then produced for maximising the proposal's positive health effects and minimising its negative health effects. Other examples include deliberative approaches, such as citizens jury. The citizens jury process is a comprehensive tool that allows decision makers to hear thoughtful citizen input. It provides an opportunity for citizens to learn about an issue, deliberate together over a period of 4–5 days, and develop well-informed solutions to difficult policy issues [61].

**Box 5a. A series of 3D animations of gears showing how gears (researchers and decision makers) can engage each other in a large number of different ways [57]**

Available from: http://commons.wikimedia.org/wiki/Category:Animations_of_gears_and_gearboxes

**Description:** Category: animations of gears and gearboxes

**Box 5b. A 3D animation showing how a third gear (a knowledge broker) can make two engaging gears (a researcher and a policy maker) turn in the same direction[58]**

Available from: http://www.technologystudent.com/gears1/gears2.htm

**Description:** An ‘idler’ gear is another important gear. In the example below gear ‘A’ turns in an anticlockwise direction and also gear ‘C’ turns in an anticlockwise direction. The ‘idler’ gear is used so that the rotation of the two important gears is the same.

**Box 5c. A 3D animation showing why the linear speed at the outer parts of two gears (a fast rotating small gear and a slow rotating large gear) must be the same [59]**

Available from: http://en.wikipedia.org/wiki/Gear

**Description:** Two meshing gears transmitting rotational motion. Note that the smaller gear is rotating faster. Although the larger gear is rotating less quickly, its torque is proportionally greater. One subtlety of this particular arrangement is that the linear speed at the pitch diameter is the same on both gears.

#### 3.3.8 Building the future based on lessons learned from the past

Summarizing the lessons learned from a review of historical perspective of epidemics from the past 5,000 years, Choi and Pak proposed 12 future challenges for public health surveillance in the 21^st^ century to: (1) expand the current surveillance system to include besides deaths also new cases for diseases; (2) develop long-term plans for surveillance systems and to avoid ad hoc systems; (3) develop ground rules on when and how to add or delete, or change the definitions of, variables under surveillance when new scientific evidence arises; (4) develop large scale and widespread data collection systems which are population-based; (5) expand the current surveillance system based mainly on health outcomes to also include risk factors and intervention indicators; (6) develop novel analysis tools and new statistics to facilitate development of disease prevention and control strategies; (7) develop surveillance systems that are closely integrated with etiologic research; (8) develop better and more accurate methods for forecasting; (9) develop a more direct and effective mechanism to feed information into the public health decision making process; (10) develop better evaluation protocols for public health programs and intervention using surveillance data; (11) develop better ways of dissemination of information to all those who need to know; (12) ensure that the surveillance system would achieve health for all, on an equal basis and without prejudice [4].

Since the Choi and Pak paper of 2001 [4], there have been rapid developments in information technology. Two more lessons are learned from the recent past. The first is the use of social media. Social media include websites and applications that enable users to create and share content or to participate in social networking. Use of social media opens up the possibility of tracking positive health [62]. This is not possible using traditional surveillance methods (such as deaths and hospitalizations). Twitter has been used to track happiness of 2.4 million people in 84 countries [63]. The second is the use of big data. Big data refers to extremely large data sets that may be analyzed computationally to reveal patterns, trends, and associations, especially relating to human behavior and interactions. Typically, big data consist of billions to trillions of records of millions of people, all coming from different sources. While big data can become the next frontier for innovation, it has associated challenges such as data storage and analysis and a host of others that need to be resolved [26].

#### 3.3.9 Building surveillance capacity

There are current deficiencies and unevenness in the surveillance capacity that will need to be improved. To avoid fragmentation in national surveillance efforts, there is a need for federal agencies to provide national facilitation to foster interstate and inter-county collaboration [24]. Central guidance can lead to coordination across states and counties, interstate technology transfer, and opportunity to learn from the successes and failures of other localities [64]. No attempt to meet the current challenges in public health surveillance will succeed unless it recognizes the fundamental importance of providing and maintaining a cadre of highly trained and motivated public health professionals in every local health agency in the country [65]. Public health surveillance systems must be strengthened by: (1) allocating resources, including human resources, for the effective use of health surveillance data and tools, and (2) recognizing the need for existing staff to acquire new skills [53].

It is not an easy task to build capacity and to tackle the unevenness in global capacity. Seven themes have been suggested to build surveillance capacity, concisely summarised by the acronym “SCIENCE”: Strategy, Collaboration, Information, Education, Novelty, Communication and Evaluation (Table 4) [66].

**Table 4.** Seven themes to build surveillance capacity, concisely summarised by the acronym “SCIENCE” [66].

Acronym “SCIENCE”	Theme	Description
**S**	Strategy	Develop a strategy to promote and market chronic disease surveillance, prevention and control.
**C**	Collaboration	Involve multiple stakeholders from all walks of society in devising a comprehensive approach to surveillance, prevention and control.
**I**	Information	Improve accuracy, timeliness, accessibility and global comparability of surveillance information to develop policies and programs.
**E**	Education	Inform scientists, policy-makers and the public about the current epidemiological shift from infectious to chronic diseases, and the importance of preventing these problems.
**N**	Novelty	Develop novel ways of thinking about both traditional and emerging problems.
**C**	Communication	Develop effective ways to convey chronic disease messages and the results and findings from surveillance to key audiences, such as policy-makers and the general public, who generally do not read scientific publications.
**E**	Evaluation	Assess the design, implementation, utility and effectiveness of initiatives in chronic disease, with emphasis on ensuring that these efforts produce public health benefits.

#### 3.3.10 Enhancing global public health surveillance

Public health surveillance must be coordinated on a global scale. This is obvious for infectious disease surveillance. Globalization of trade and the economy has resulted in a constant massive mobilization of commodities and people across countries and continents at unprecedented speed. It takes only a few hours to transport or mobilize thousands of people and goods across the globe. It is possible to travel between most places in the world in less time than the incubation period for many infectious diseases [67]. What is less obvious is that there is also a need for global surveillance for risk factors for chronic diseases. While chronic diseases are not transferable, their risk factors are. International migrants bring with them their cooking styles, hygiene practices, etc., thereby affecting both the infectious and chronic disease patterns in the host country [68]. In this sense, chronic non-communicable diseases like cardiovascular diseases can be considered communicable [69].

In global chronic disease surveillance, new global health surveillance networks have emerged. Examples include the World Alliance for Risk Factor Surveillance (WARFS) [70],[71], and the Americas' Network for Chronic Disease Surveillance (AMNET) [72],[73] (Table 5). WARFS is the Global Working Group on Surveillance of the International Union for Health Promotion and Education (IUHPE). It supports the development of behavioural risk factor surveillance as a tool for evidence-based public health, acknowledging the importance of this information source to inform, monitor and evaluate disease prevention and health promotion policies, services and interventions [70]. There has been a series of biennial global conferences on risk factor surveillance, beginning in USA (Atlanta), 1999; Finland (Tuusula), 2001; Australia (Noosaville), 2003; Uruguay (Montevideo), 2005; Italy (Rome), 2007; Italy (Venice), 2009; Canada (Toronto), 2011; and China (Beijing), 2013. AMNET was established in 2003 as a regional network for the purposes of sharing information and experiences, as well as providing opportunities for enhancing chronic disease surveillance in the WHO Region of Americas (North, Central and South America, and the Caribbean) [72]. Both networks have made significant contributions consistently over a long period to global and regional (Region of Americas) public health surveillance, respectively, and are expected to make further contributions in the future. The International Association of National Public Health Institutes (IANPHI) builds national institutes of public health, all of which have surveillance function at its core [74].

**Table 5.** Two international chronic disease surveillance networks—World Alliance for Risk Factor Surveillance (WARFS) and Americas' Network for Chronic Disease Surveillance (AMNET).

Name	Description
**World Alliance for Risk Factor Surveillance (WARFS) (Since 1999)**	WARFS is the Global Working Group on Surveillance of the International Union for Health Promotion and Education (IUHPE). It supports the development of behavioural risk factor surveillance (BRFS) as a tool for evidence-based public health, acknowledging the importance of this information source to inform, monitor and evaluate disease prevention and health promotion policies, services and interventions [71].Official website: http://www.warfs.info/
**Americas' Network for Chronic Disease Surveillance (AMNET) (Since 2003)**	AMNET promotes chronic disease surveillance, translating knowledge into action, building public health capacity in the Region of the Americas (North, Central and South Americas and the Caribbean). It promotes the integration of governmental and nongovernmental organizations that advances training in surveillance and prevention of chronic disease in American population [72].Official website: http://www.redamnet.org/

## 4. Discussion

The importance of strengthening public health surveillance to provide early warning and develop actions has been a primary focus in public health. However, despite improvements in the past decades, public health surveillance capabilities remain limited and fragmented, with uneven global coverage. Some future scenarios are presented: exploring new frontiers; enhancing computer technology; improving epidemic investigations; improving data collection, analysis, dissemination and use; building on lessons from the past; building capacity; and enhancing global surveillance. It must be pointed out that the list of future scenarios is based on a review of the current literature, and does not represent a complete list of future possibilities. Also, while some scenarios are applicable to certain countries, they may neither be applicable to all countries, nor at all times.

This article is intended to raise questions and stimulate a debate on the important topic of where public health surveillance will go from here. Lao-tzu (604 BC-531 BC), a Chinese philosopher, once said, “*A journey of a thousand miles begins with a single step*” [75]. Let us continue to use our creative and innovative thinking to inform our next steps in public health surveillance.

## 5. Conclusion

It is hoped that learning from the past, reflecting on the present, and planning for the future can further enhance public health surveillance for the good of humankind.
